# Starmerella fangiana f.a. sp. nov., a new ascomycetous yeast species from Daqu-making environment and other sources

**DOI:** 10.1099/ijsem.0.006581

**Published:** 2024-11-20

**Authors:** Yu-Hua Wei, Hai-Yan Zhu, Zhang Wen, Liang-Chen Guo, Mei Bai, Di-Qiang Wang, Wei Huang, Li-Li Jiang, Napapohn Kajadpai, Nantana Srisuk, Pei-Jie Han, Feng-Yan Bai

**Affiliations:** 1 State Key Laboratory of Mycology, Institute of Microbiology, Chinese Academy of Sciences, Beijing 100101, PR China; 2 College of Life Sciences, University of Chinese Academy of Sciences, Beijing 100049, PR China; 3 College of Life Science, Key Laboratory of Medicinal Chemistry and Molecular Diagnosis of Ministry of Education, Hebei University, Baoding 071002, PR China; 4 GuiZhou XiJiu Co., Ltd, Guizhou 564622, PR China; 5 Department of Microbiology, Faculty of Science, Kasetsart University, Bangkok 10900, Thailand; 6 Biodiversity Center Kasetsart University, Bangkok 10900, Thailand

**Keywords:** ascomycetous yeast, phylogeny, *S. fangiana *f.a. sp. nov, taxonomy

## Abstract

In the survey of yeast diversity in high-temperature Daqu, which is a fermentation starter for Chinese sauce-flavoured Baijiu, six yeast strains representing one novel species of the genus *Starmerella* were isolated from samples of Daqu and surrounding environments collected in Zunyi city, Guizhou Province, China. Phylogenetic analyses of the internal transcribed spacer (ITS) region and the D1/D2 domains of the large subunit rRNA gene indicate that these six strains are conspecific with three other strains isolated from flowers and duckweed collected in Samoa, India and Thailand. The representative strain QFC-8 of the new species differs from the closet species *Starmerella caucasica* resolved by the D1/D2 sequence analysis by 13 (3.1 %, 12 substitutions and 1 gap) and 40 (10.3 %, 9 substitutions and 31 gaps) mismatches in the D1/D2 domain and ITS region, respectively. The results suggest that the novel group represents an undescribed species in the genus *Starmerella*, for which the name *Starmerella fangiana* sp. nov. is proposed. The holotype strain is CGMCC 2.7773.

## Introduction

The genus *Starmerella* was proposed by Rosa and Lachance in 1998 to accommodate the sexual stage of *Candida bombicola* [1]. *Starmerella bombicola* (syn: *Candida bombicola*) formed a highly supported clade together with *Candida etchelsii*, *Candida bombicola*, *Candida apicola*, *Candida floricola*, *Candida stellata* and *Candida lactis-condensis*. This clade was closely related to a sister clade including five other *Candida* species based on D1/D2 sequences analysis, implying that these two clades represent two different genera [2]. Subsequently, several species without a known sexual state were described as members of the genus *Starmerella*, including *Starmerella aceti*, *Starmerella anomalae*, *Starmerella asiatica*, *Starmerella caucasica*, *Starmerella gilliamiae*, *Starmerella henanensis*, *Starmerella jinningensis*, *Starmerella monicapupoae*, *Starmerella neotropicalis*, *Starmerella orientalis*, *Starmerella scarabaei* and *Starmerella syriaca* [3,10]. Likewise, Santos *et al*. described 6 others novel *Starmerella* species without a known sexual state and transferred 25 *Candida* species to the genus *Starmerella* according to the one fungus, one name concept implemented in the *International Code of Nomenclature for algae, fungi, and plants* [11,12]. Currently, 51 species are included in the genus *Starmerella*. Among the *Starmerella* species described so far, only two species, *Starmerella bombicola* and *Starmerella meliponinorum*, have a sexual state that produces conjugate asci with a single, roughened and asymmetrical ascospore [1,13].

Daqu, which is a brick-shaped product fermented spontaneously from wheat, barley and beans, is an essential starter for Baijiu fermentation [14]. It provides the majority of functional micro-organisms, enzymes and flavour precursors for Baijiu fermentation [15]. Daqu is mainly classified into three types based on the maximum temperature reached during the fermentation process: low-temperature (40–50 °C), medium-temperature (50–60 °C) and high-temperature (60–70 °C) Daqu. Among them, high-temperature Daqu serves as the starter for producing sauce-flavour Baijiu. Since Daqu is fermented from non-sterile grains in an open environment, the micro-organisms from raw grains and associated environments contribute to the microbial community enriched in Daqu.

During an investigation of yeast diversity in the fermentation process and related environments of high-temperature Daqu, we found that six yeast strains isolated from samples of Daqu and surrounding workshop environments represent a novel species of the genus *Starmerella*, together with three strains previously isolated from other different substrates collected in Samoa, India and Thailand.

## Sampling

Samples of high-temperature Daqu with different fermentation stages and dusts on doors and windows of Daqu-making workshops were collected in Zunyi city, Guizhou Province, China in August 2023. Daqu samples were placed into sterile bags. Dust samples from Daqu workshop doors and windows were collected with sterile cotton swabs soaked with 0.1 M PBS buffer. An area of 0.2 m^2^ was wiped, and the swab was placed into a sterile 50 mL centrifuge tube. Samples were transferred to the laboratory at 4 °C ice box and subjected to yeast isolation immediately.

## Yeast isolation

For Daqu samples, 10 g of a sample was suspended in 90 mL sterile water in a 250 mL sterile conical flask. For the dust samples from doors and windows, ~15 mL sterile water was added to the 50-mL centrifuge tube to completely cover the cotton swabs. Subsequently, each suspension was diluted to 10^−2^ and 10^−3^, and 100 µL of each dilution was plated on YPD agar plates supplemented with 200 µg mL^−1^ chloramphenicol and 2% acetic acid. After the incubation at 30 °C for 3 days, yeast colonies with distinct morphological features were picked and purified for further study. The purified yeast strains were suspended in 20% (v/v) glycerol and stored at −80 °C.

## Phenotypic characterization

Morphological, biochemical and physiological characterizations were performed using the methods described by Kurtzman *et al*. [16]. The formation of pseudohyphae was observed microscopically after inoculating the strain at 25 °C for 1 month on potato dextrose agar (PDA) (20% potato infusion, 2% glucose and 2% agar) and corn meal agar (CMA) (2.5% corn starch and 2% agar) with a cover glass covering the colony to create an oxygen-free environment. Carbon and nitrogen compound assimilation tests were conducted in liquid medium. Fermentation tests were done with Durham inverted tubes [16]. The growth at different temperatures was conducted on YPD agar. The formation of sexual structures was investigated for individual strain and pairwise mixed strains on yeast carbon basal agar (YCB) (1.17% yeast carbon base and 2% agar), CMA, PDA and V8 agar (10 % V8 juice and 2% agar) incubated at 25 °C for up to 2 months and examined periodically.

## Molecular phylogenetic analyses

Genomic DNA was extracted according to the method described by Wang and Bai [17]. The internal transcribed spacer (ITS) region (including 5.8S rDNA) and the D1/D2 domains were amplified and sequenced using primer pairs ITS1-ITS4 [18] and NL1-NL4 [19], respectively. Yeast strains were primarily identified using the blast search through GenBank with their ITS and D1/D2 sequences as queries [20]. Sequences of closely related strains or species were retrieved from GenBank and were aligned using MAFFT v.7 [21]. Positions that were ambiguous to align were excluded manually. Phylogenetic trees were constructed from the evolutionary distance data with Kimura’s two-parameter model using the neighbour-joining method in mega v.7 [22,24], and the maximum likelihood analyses based on the combined ITS and D1/D2 sequences were performed using RAxML v.8 [25]. The confidence levels of the clades were estimated through bootstrap analysis with 1000 replicates [26].

Among the yeast strains isolated in this study, six strains including strain D5S-2 isolated from a sample of Daqu fermented for 5 days; strains QFC-8, QFC-Y-4 and QFC-Y-6 from dusts sampled from windows; and strains QFM-Y-5 and QFM-Y-10 from dusts sampled from doors of the Daqu-making workshop (Table 1) share identical or similar (only one indel or one base substitution difference) sequences in both the D/D2 domain and the ITS region, suggesting that they are conspecific. The D1/D2 sequence blast results through the GenBank database indicated that the six strains represented by strain QFC-8 isolated in this study possess identical or similar (one to two base mismatches) D1/D2 sequences with eight strains from other sources, including strains F10, PL0702 and ST1-01 isolated from honey bee in Thailand, strain DMKU-DWEN32-1 isolated from duckweed in Thailand, strains 16S1 and HSB-14 isolated from inflorescence of *Ruellia* sp. and honey bee in India, strain IMT-O from an unknown substrate in India and strain 11–1463 isolated from flower in Samoa (Table 1). Three of the eight foreign strains (DMKU-DWEN32-1, 16S1 and 11–1463) have ITS sequences available for comparison, and they are either identical to the Chinese strains or differ by two substitutions and one to three gaps in the ITS region (Table 1). Two additional Indian strains (CHF-11E and IMT-E) from unknown sources were found to possess identical ITS sequences with the Chinese strains from the result of the ITS sequence blast search (Table 1), suggesting that they may also belong to the same species as the Chinese strains.

**Table 1.** Strains of *Starmerella fangiana* sp. nov. and closely related species and their sequence differences from the type strain of the new species

Species	Strain	Origin	Source	D1/D2 domain			ITS region		
				Accession no.	Total length (bp)	Nucleotide substitution/	Accession no.	Total length	Nucleotide substitution/
						gaps			gaps
*S. fangiana* sp. nov.	QFC-8	China	Dusts on windows of Daqu-making workshops	PP786676	422		PP786670	397	
	= CGMCC 2.7773^T^								
	QFM-Y-5	China	Dusts on doors of Daqu-making workshops	PP786673	422	0/0	PP786667	397	0/0
	QFM-Y-10	China	Dusts on doors of Daqu-making workshops	PP786674	422	0/1	PP786668	397	1/0
	D5S-2	China	High-temperature Daqu	PP786675	422	0/1	PP786669	397	0/0
	QFC-Y-4	China	Dusts on windows of Daqu-making workshops	PP786677	422	0/1	PP786671	397	0/0
	QFC-Y-6	China	Dusts on windows of Daqu-making workshops	PP786672	422	0/1	PP786666	397	0/0
	DMKU-DWEN32-1	Thailand	Duckweed	ON065764	422	0/0	PQ323321	393	0/0
	11–1463	Samoa	Flower	MF375635	422	1/0	MG594032	362	2/1
	16 S1	India	Inflorescence of *Ruellia* sp.	AM931020	422	0/0	FN545853	333	2/3
*Starmerella* sp.	F10	Thailand	Digestive tract of red dwarf honey bee	LC434105	422	1/0	Not available	Not available	Not available
*Starmerella* sp.	PL0702	Thailand	Honey of red dwarf honey bee	LC487601	422	2/0	Not available	Not available	Not available
*Starmerella* sp.	ST1-01	Thailand	Honey bee	AB294245	422	0/0	Not available	Not available	Not available
*Starmerella* sp.	HSB-14	India	Honey bee surface (*Apis cerana*)	MN832583	422	0/0	Not available	Not available	Not available
*Starmerella* sp.	IMT-O	India	Unknown	MN832571	422	0/0	Not available	Not available	Not available
*Starmerella* sp.	CHF-11E	India	Unknown	Not available	Not available	Not available	MN861622	394	0/0
*Starmerella* sp.	IMT-E	India	Unknown	Not available	Not available	Not available	MN832566	394	0/0
*Starmerella riodocensis*	CBS 10087^T^	Thailand	Honey bee	KJ630496	424	20/1	NR 137870	397	8/24
*S. caucasica*	CBS 12650^T^	Azerbaijan	Flowers of *Wisteria sinensis*	JX112043	422	12/1	KY105545	390	9/31

**Fig. 1.**
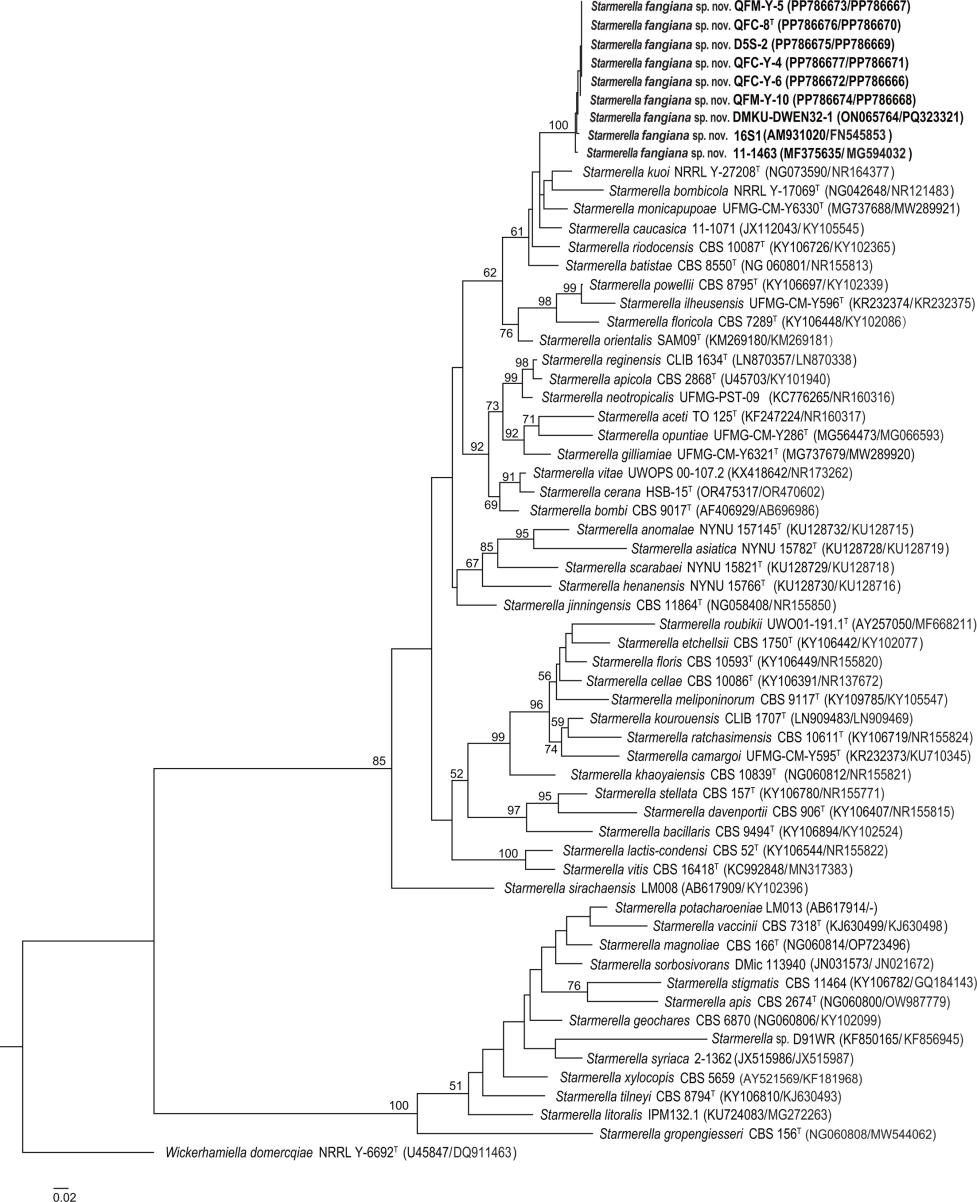
Maximum likelihood phylogenetic tree based on the combined sequences of the D1/D2 domain and the ITS region showing the phylogenetic placement of the novel species *Starmerella fangiana* sp. nov. Bootstrap values ≥50% are shown on the branches of the tree. Species *Wickerhamiella domercqiae* is used as the outgroup. Type strains are denoted with a superscripted ‘T’. Bar, 0.02 substitutions per nucleotide position.

The phylogenetic tree constructed based on the D1/D2 sequences showed that the six Chinese strains and the eight strains from other countries form a branch which is most closely related to *S. caucasica* (Fig. S1, available in the online Supplementary Material). The representative Chinese strain QFC-8 differs from the type strain of *S. caucasica* by 13 (3.1 %, 12 substitutions and 1 gap) and 40 (10.3 %, 9 substitutions and 31 gaps) mismatches in the D1/D2 domain and ITS region, respectively. The phylogenetic tree constructed using the ITS sequences shows that the six Chinese strains and the five foreign strains with ITS sequences available for comparison form a well-separated branch in the *Starmerella* clade, but the phylogenetic relationship of this group with the other species of the genus is not resolved in the ITS tree (Fig. S2). A blast search through the GenBank database with the ITS sequence of strain QFC-8 as the query showed that the top match was *Starmerella riodocensis*. Strain QFC-8 differs from the type strain of *S. riodocensis* by 21 (5.0 %, 20 substitutions and 1 gap) and 32 (8.1 %, 8 substitutions and 24 gaps) mismatches in the D1/D2 domain and the ITS region, respectively. The phylogenetic analysis based on the combined D1/D2 and ITS sequences revealed that the six Chinese strains and the three foreign strains (DMKU-DWEN32-1, 16S1 and 11–1463) with both D1/D2 and ITS sequences being available were clustered together in a distinct group which was clearly separated from the other species of the genus *Starmerella*; however, the phylogenetic position of the new group was not statistically supported (Fig. 1).

The phylogenetic analyses suggest that the six Chinese strains isolated Daqu and Daqu-making environment are conspecific with three other foreign strains (11–1463, 16S1 and DMKU-DWEN32-1) from flowers and duckweed with both D1/D2 and ITS sequences being available for comparison (Table 1). These nine strains represent one novel species in the genus *Starmerella*, for which the name *Starmerella fangiana* sp. nov. is proposed. The other seven foreign strains with identical or similar D1/D2 or ITS sequences with the Chinese strains (Table 1) are also most possibly belong to the new species.

## Phenotypical characteristics and ecology

The morphological, biochemical and physiological characteristics of the six Chinese strains isolated from Daqu and related environment and strain DMKU-DWEN32-1 from duckweed collected in Thailand were determined. Physiologically, *S. fangiana* differs from its closely related species *S. caucasica* by its abilities to grow at 40 °C and assimilate succinic acid, ethylamine and cadaverine [5] and differs from its another closely related species *S. riodocensis* by its positive growth at 40 °C and assimilate sucrose, raffinose, ribose, ethanol, succinic acid, xylitol and nitrate [27]. The sexual state of the new species was not observed in the single and pairwise mixed strain cultures of the six Chinese strains and the Thai strain DMKU-DWEN32-1 on different media.

The majority of the known *Starmerella* species are from bees and associated flowers [1,5,8, 11, 13, 27], with a few species being isolated from other insects or beverages, such as scarabs beetles [4], leaf-cutting ant [3], concentrated grape juice [32] and fermented sweet botrytized wines [33]. The species from the sources other than insects also likely have an affinity with insects which may transfer the species to various high-sugar substrates that they visit. The new species *S. fangiana* described in this study is also most likely associated with insects. Among the strains of *S. fangiana* sp. nov. compared in this study with known sources, six Chinese strains were from Daqu fermentation process and related environments, and seven foreign strains originated from bees, flowers and duckweed (Table 1). Although the Chinese strains were not directly isolated from insects, their relationship with insects is expected. Daqu is produced through spontaneous solid-state fermentation of wheat in an open environment. Insects, mainly Coleoptera beetles, frequently occur in Daqu bricks and the workshop environment. This implies a possible affinity of the Chinese strains of the novel species with insects.

## Description of *Starmerella fangiana* Y.H. Wei, P.J. Han, D.Q. Wang & F.Y. Bai sp. nov.

*Starmerella fangiana* (fang.i.a’na. N.L. fem. adj. *fangiana*, of Fang, named in honour of Xinfang Fang for his pioneering contributions to the study of yeasts associated with Daqu and Baijiu fermentation in China).

**Fig. 2.**
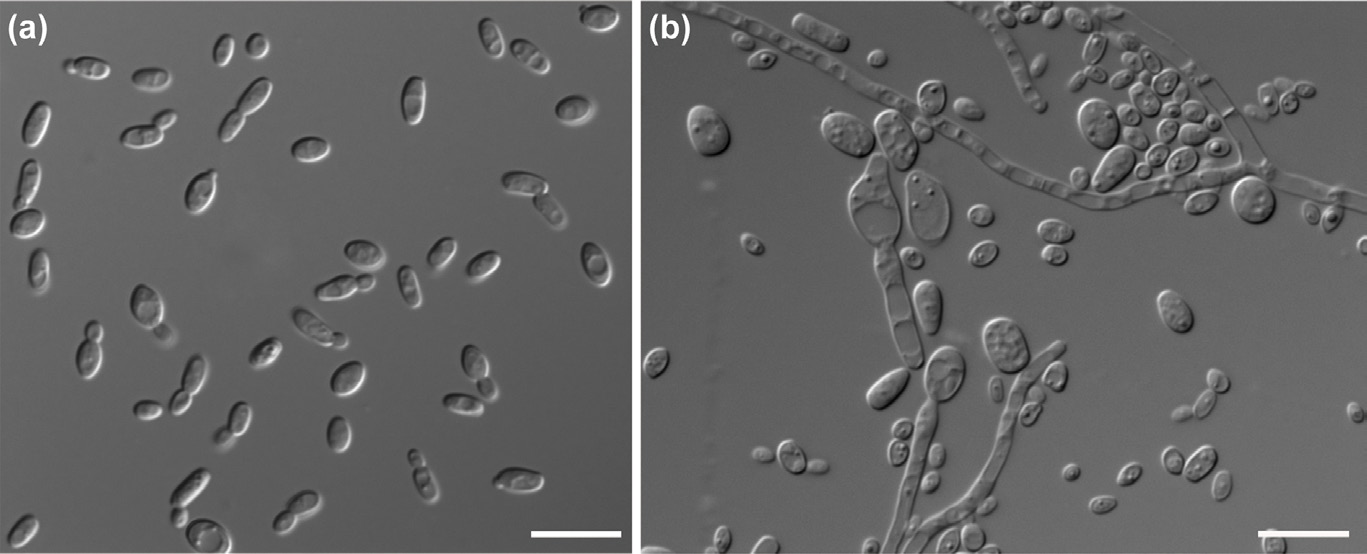
Morphology of *Starmerella fangiana* sp. nov. (**a**) Vegetative cells of strain QFC-8^T^ on YM agar after 3 days at 25 °C. (**b**) True hyphae of strain D5S-2 on PDA agar after 1 month at 25 °C. Bars, 10 µm.

Culture characteristics: After growth on yeast extract–malt extract (YM) agar for 1 week at 25 °C, the cells are ovoid to ellipsoidal (2.1–4.0×2.8–6.1 µm) and occur singly or as budded pairs; reproduction is by multilateral budding (Fig. 2a). On YM agar after 3 days at 25 °C, colonies are cream, opaque and smooth with the entire margin. After growth in YM broth for 1 month at 25 °C, ring and sediment are formed. The true hyphae of strain D5S-2 formed on Dalmau plates after 1 month on PDA agar at 25 °C (Fig. 2b). Pseudohyphae are not observed on Dalmau plates after 1 month on PDA agar and CMA agar at 25 °C. Asci or signs of conjugation are not observed on YCB, CMA, PDA and V8 after 2 months at 25 °C.

Physiological and biochemical characteristics: Glucose and sucrose fermentation are strong or delay. But trehalose, galactose, maltose, lactose and raffinose are not fermented. Glucose, d-galactose (or delay), l-sorbose, sucrose, raffinose, d-ribose (or weak), ethanol, glycerol, mannitol, glucitol, succinic acid (weak), xylitol (or weak) and citric acid (weak) are assimilated as sole carbon sources. d-maltose, d-cellobiose, trehalose, lactose, melibiose, melezitose, inulin, starch soluble, d-xylose, l-arabinose, d-arabinose, l-rhamnose, d-glucosamine, methanol, erythritol, ribitol, galactitol, α-methyl-d-glucoside, salicin, d-glucuronic acid, dl-lactic acid, inositol, hexadecane and N-acetyl-d-glucosamine are not assimilated as sole carbon sources. Ethylamine, cadaverine, ammonium sulphate, l-lysine and potassium nitrate (or delay) are assimilated as sole nitrogen sources. Sodium nitrite is not assimilated as sole nitrogen sources. Urease activity is negative. Diazonium blue B reaction is positive. Extracellular starch compounds are not produced. Growth in 10% (w/v) sodium chloride plus 5% (w/v) glucose medium is positive. Growth on 50% (w/v) glucose–yeast extract agar and 60% (w/v) glucose–yeast extract agar is positive. Growth in vitamin-free medium is positive. Growth on YPD agar is positive at 40 °C but negative at 45 °C.

The holotype, CGMCC 2.7773 (original strain number QFC-8), was isolated from dusts on windows of Daqu-making workshops collected in Zunyi City, Guizhou Province, China, in August 2023 and has been deposited in a metabolically inactive state in the China General Microbiological Culture Collection Center (CGMCC), Beijing, PR China. The ex-type culture has been deposited in the Japan Collection of Microorganisms (JCM), Koyadai, Japan, as JCM 36912. The GenBank/EMBL/DDBJ accession numbers for the sequences of the D1/D2 domain and the ITS region of strains QFC-8, QFM-Y-5, QFM-Y-10, D5S-2, QFC-Y-4 and QFC-Y-6 are PP786676, PP786673, PP786674, PP786675, PP786677 and PP786672 and PP786670, PP786667, PP786668, PP786669, PP786671 and PP786666, respectively. The fungal name number is FN 571937.

## supplementary material

10.1099/ijsem.0.006581 Uncited Fig. S1.

## Abbreviations

CGMCC: China General Microbiological Culture Collection Center
CMA: corn meal agar
ITS: internal transcribed spacer
JCM: Japan Collection of Microorganisms
PDA: potato dextrose agar
YCB: yeast carbon basal agar
YM: yeast extract–malt extract
YPD: yeast extract peptone dextrose
