# Supplementary material for: Starmerella fangiana f.a. sp. nov., a new ascomycetous yeast species from Daqu-making environment and other sources
Source: Int J Syst Evol Microbiol. 2024 Nov 20;74(11):006581. doi: 10.1099/ijsem.0.006581 (PMC11578291; doi:10.1099/ijsem.0.006581)
Supplement: Uncited Fig. S1. [file ijsem-74-06581-s001.pdf]

Supplementary materials

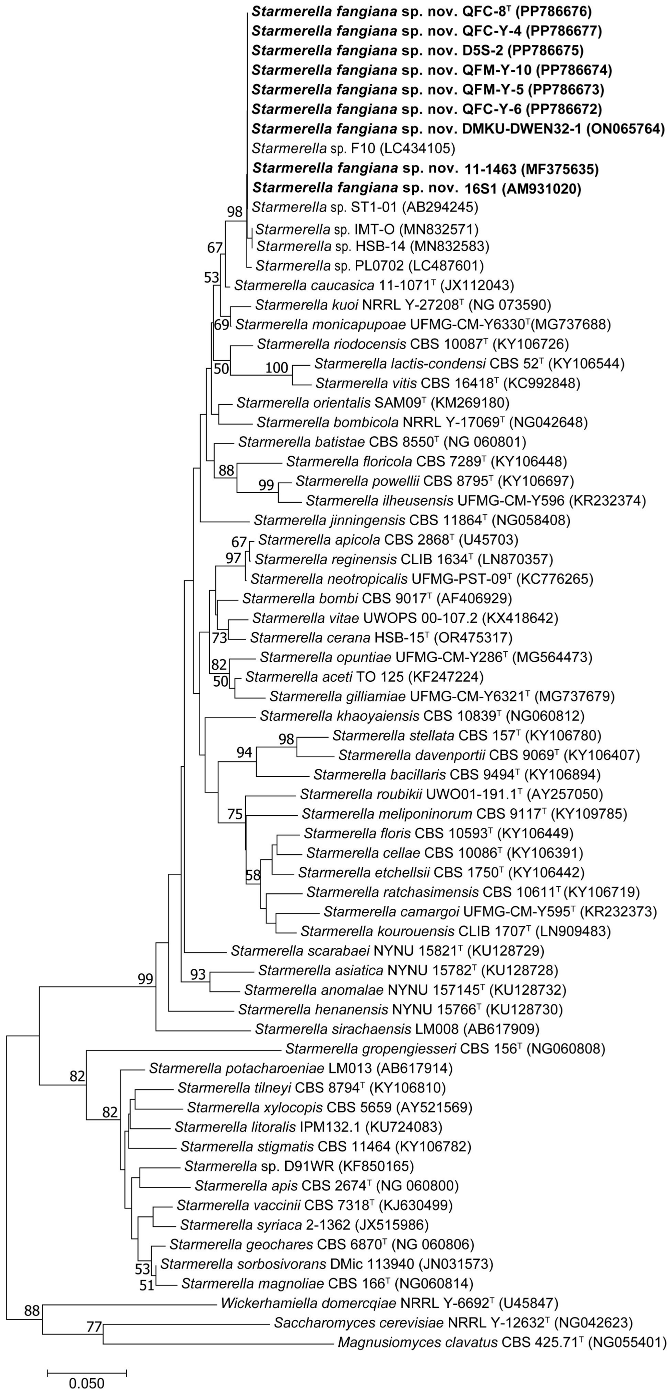

Fig. S1 Neighbor-Joining phylogenetic tree based on the D1/D2 domain showing the phylogenetic placement of the novel species *Starmerella fangiana* sp. nov. Bootstrap values ≥ 50% are shown on the branches of the tree. Species *Wickerhamiella domercqiae*, *Saccharomyces cerevisiae* and *Magnusiomyces clavatus* are used as the outgroup. Type strains are denoted with a superscripted ‘T’. Bar, 0.05 substitutions per nucleotide position.

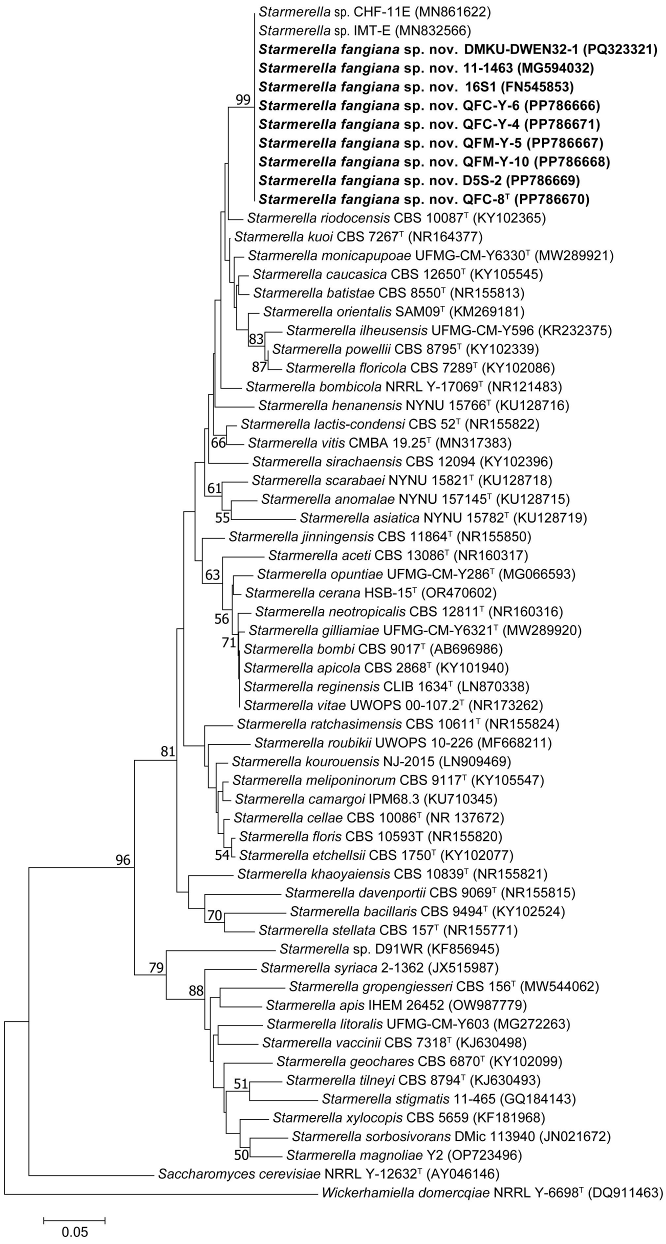

Fig. S2 Neighbor-Joining phylogenetic tree based on the ITS region showing the phylogenetic placement of the novel species *Starmerella fangiana* sp. nov. Bootstrap values ≥ 50% are shown on the branches of the tree. Species *Saccharomyces cerevisiae* and *Wickerhamiella domercqiae* are used as the outgroup. Type strains are denoted with a superscripted 'T'. Bar, 0.05 substitutions per nucleotide position.
